# Quasi-Targeted Metabonomics Reveals Metabolites Associated with Antioxidant Activity of *Mesona chinensis* Benth Cultivar Xiaoye

**DOI:** 10.3390/plants14111585

**Published:** 2025-05-23

**Authors:** Yuqing Niu, Meixia Zheng, Dagang Tian, Yanming Zhu, Hong Chen, Yujing Zhu, Hailan Su

**Affiliations:** 1 Institute of Crop Sciences, Fujian Academy of Agricultural Sciences, Fuzhou 350013, China; niuyuqing804@163.com (Y.N.); zhengmeixia2005@163.com (M.Z.); mumuzym@163.com (Y.Z.); chenhong6313@163.com (H.C.); 2 Biotechnology Research Institute, Fujian Academy of Agricultural Sciences, Fuzhou 350013, China; tdg@fjage.org

**Keywords:** *M. chinensis* Benth, Xiaoye variety, secondary metabolites, antioxidant activity, UHPLC-MS

## Abstract

*Mesona chinensis* Benth is a significant botanical resource utilized for both medicinal and dietary purposes, and the Xiaoye variety (XY) exhibited the highest antioxidant activity among the varieties. Despite its importance, metabolic information regarding its medicinal and nutritional properties remains sparse. This study examined the secondary metabolites of four *M. chinensis* Benth varieties using UHPLC-MS/MS and identified 102, 105, and 286 metabolites exhibiting differential accumulation in the XY variety compared to the Taiwan variety (TW), Minxuan variety (MX), and Zengcheng variety (ZC), respectively, among the 1287 metabolites identified. These metabolites are predominantly involved in secondary metabolic pathways such as “Tropane, Piperidine, and Pyridine Alkaloid Biosynthesis” and “Flavone and Flavonol Biosynthesis”. In addition, we identified the ten most significant differential metabolites that influence antioxidant activity, with flavonoids recognized as the primary contributors to the variation in antioxidant activities. In this study, we have outlined the metabolic landscape of *M. chinensis* Benth. These findings may aid in elucidating the mechanism behind the antioxidant activity of XY, which provides valuable insights for breeding, quality assurance, and product innovation related to *M. chinensis* Benth.

## 1. Introduction

*Mesona chinensis* Benth, a perennial herb of the Lamiaceae, is native to southern China and several Southeast Asian countries. This plant has a long history of cultivation and use [1]. Studies have shown that extracts from *M. chinensis* Benth possess potent antioxidant, anti-hypoxia, anti-hypertension, and anti-lipid properties [2,3,4,5], Its primary bioactive compounds include polysaccharides, flavonoids, phenols, and other chemical constituents [6]. The levels of flavonoids, total phenols, and related compounds significantly contribute to the plant’s antioxidant properties, but they vary among different varieties [7,8]. These antioxidant properties primarily involve neutralizing free radicals and are associated with reducing oxidative stress linked to conditions such as diabetes and atherosclerosis [9,10,11]. Furthermore, *M. chinensis* Benth is a key ingredient in contemporary herbal tea beverages and medicinal powders, highlighting its significant culinary and medicinal value.

By screening varieties with strong antioxidant activity and comparing the metabolite differences between those with high antioxidant activity and other varieties, we can uncover the metabolic characteristics and biosynthetic pathways of the high-antioxidant varieties, which can inform the molecular breeding of *M. chinensis* Benth. Previous research has focused mainly on the qualitative and quantitative analysis of a limited range of metabolites, without simultaneously considering the quantity and relative content of these substances [12]. Quasi-targeted metabonomics is an innovative metabolome detection technology that merges the high throughput of non-targeted metabolomics with the precision and sensitivity of targeted metabolomics. This technology utilizes a triple quadrupole linear ion trap composite SCIEX QTRAP^®^ 6500+ mass spectrometer and employs Multiple Reaction Monitoring (MRM) to accurately characterize and quantify metabolites in biological samples. Ultra-high performance liquid chromatography (UHPLC-MS) combines the efficient separation capabilities of UHPLC with the sensitive and selective detection of mass spectrometry, facilitating rapid and precise analysis of trace compounds in complex samples. At present, quasi-targeted metabonomics is widely utilized in drug development, metabolomics, environmental monitoring, and food safety, providing high throughput and cost-effective benefits, making it ideal for analyzing trace yet biologically active compounds in *M. chinensis* Benth, such as polyphenols, flavonoids, organic acids, sugars, etc. [13,14].

Herein, we employed the quasi-targeted metabonomics approach to explore the metabolic changes linked to antioxidant activities in four well-characterized varieties. Additionally, we examined the correlations of various metabolites (such as flavonoids and lipids) with the levels of antioxidant properties. Our aim was to identify the primary contributors to variations in antioxidant activities and the corresponding metabolic pathways that could enhance our understanding of the biochemical characteristics involved in the XY variety.

## 2. Materials and Methods

### 2.1. Material Collection and Preparation

**Figure 1:**
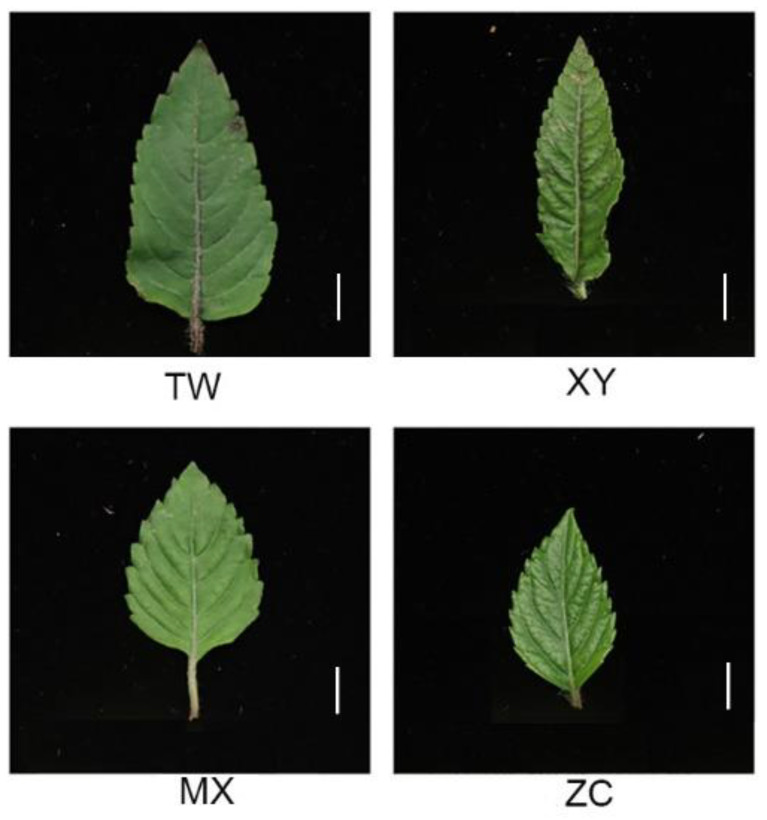
Leaves of *M. chinensis* Benth from four varieties.

Four *M. chinensis* Benth of the Taiwan variety (TW), Xiaoye variety (XY), Minxuan variety (MX), and Zengcheng variety (ZC) were cultivated in Wuping County, Fujian Province (Figure 1). In August 2023, four varieties of *M. chinensis* Benth were harvested from Wuping County, Fujian Province. Fifteen above-ground samples from each variety were pooled and preserved at −80 °C for metabolomic analysis, with three biological replicates per sample. The remaining samples were dried, ground, and sieved through a 40-mesh screen for the assessment of antioxidant activity and for determination of the content of total flavonoids, phenolic acids, and polysaccharides.

### 2.2. Determination of Antioxidant Activity

The antioxidant analysis of various varieties of *M. chinensis* Benth was performed using the alcohol extraction method. A 0.01 g sample of the powdered plant was placed into a 1.5 mL centrifuge tube, to which 1 mL of 70% ethanol was added. The mixture was then subjected to ultrasonic treatment in a water bath at 50 °C for 30 min, followed by centrifugation at 10,000 rpm for 10 min. The supernatant was collected and stored at −20 °C until analysis. The antioxidant properties were evaluated using the 2,2-Diphenyl-1-picrylhydrazyl (DPPH) method, according to the instructions provided with the kit (BC4750, Solarbio, Beijing, China), and the 2,2′-Azinobis-(3-ethylbenzthiazoline-6-sulphonate) (ABTS) method, according to the instructions provided with the kit (BC4775, Solarbio, Beijing, China), and analyzed using a microplate reader (SpectraMax Paradigm, Molecular Devices, San José, CA, USA).

### 2.3. Determination of Polyphenol Content

Mix 0.5 g of ground plant material with 10 mL of ethanol, and extract at 50 °C for 45 min. Filter to collect the polyphenol extract. Use a gallic acid standard at varying concentrations, mix with Folin–Ciocalteu reagent and sodium carbonate, and measure the absorbance at 740 nm. A standard curve was established using gallic acid concentration and absorbance values. Absorbance at 740 nm was measured for 1 mL of polyphenol extract to determine the polyphenol content in the samples, with each sample tested in triplicate.

### 2.4. Determination of Flavonoid Content

Mix 1.0 g of cold grass powder with 20 mL of 75% ethanol and ultrasonically extract at 50 °C for 45 min, then filter and dilute the extract tenfold. Prepare rutin standard solutions at concentrations of 0.2 mg/mL in volumes of 0, 1.0, 2.0, 3.0, 4.0, and 5.0 mL. Add 1.0 mL of 5% NaNO_2_ solution, wait 5 min, add 1.0 mL of 10% Al (NO_3_)_3_ solution, wait another 5 min, and finally add 10 mL of 10% NaOH solution. Adjust the volume to 25 mL with 70% ethanol and let stand for 15 min. A standard curve was created using rutin concentration on the *y*-axis and absorption at 510 nm on the *x*-axis. The regression equation was calculated. Measure absorbance at 510 nm using 5 mL of the flavonoid extract to calculate flavonoid content. Each sample was tested three times.

### 2.5. Quasi-Targeted Metabolomics

The experiment was conducted by Beijing Novogene Technology Co., Ltd. (Beijing, China). Tissues (100 mg) were individually ground with liquid nitrogen, and the homogenate was resuspended in prechilled 80% methanol by thorough vortexing. The samples were then incubated on ice for 5 min and centrifuged at 15,000× *g* at 4 °C for 20 min. A portion of the supernatant was diluted to a final concentration of 53% methanol with LC-MS grade water and transferred to a new Eppendorf tube for a second centrifugation at 15,000× *g* at 4 °C for 20 min. The supernatant was then analyzed using the LC-MS/MS system.

LC-MS/MS analyses were performed using an ExionLC™ AD system (SCIEX) coupled with a QTRAP^®^ 6500+ mass spectrometer (SCIEX) in Novogene Co., Ltd. (Beijing, China). Samples were injected onto a Xselect HSS T3 (2.1 × 150 mm, 2.5 μm) (Milford, MA, USA) using a 20 min linear gradient at a flow rate of 0.4 mL/min for the positive/negative polarity mode. The eluents were eluent A (0.1% Formic acid-water) and eluent B (0.1% Formic acid-acetonitrile) [6]. The solvent gradient was set as follows: 2% B, 2 min; 2-100% B, 15.0 min; 100% B, 17.0 min; 100-2% B, 17.1 min; 2% B, 20 min. The QTRAP^®^ 6500+ mass spectrometer was operated in positive polarity mode with Curtain Gas of 35 psi, Collision Gas of Medium, IonSpray Voltage of 5500 V, Temperature of 550 °C, Ion Source Gas of 1:60, and Ion Source Gas of 2:60. The QTRAP^®^ 6500+ mass spectrometer was operated in negative polarity mode with Curtain Gas of 35 psi, Collision Gas of Medium, IonSpray Voltage of −4500 V, Temperature of 550 °C, Ion Source Gas of 1:60, and Ion Source Gas of 2:60.

The detection of the experimental samples using MRM (Multiple Reaction Monitoring) was based on the novogene in-house database. The Q3 were used for the metabolite quantification. The Q1, Q3, RT (retention time), DP (declustering potential), and CE (collision energy) were used for the metabolite identification. The data files generated by HPLC-MS/MS were processed using SCIEX OS Version 1.4 to integrate and correct the peak. The main parameters were set as follows: minimum peak height, 500; signal/noise ratio, 5; gaussian smooth width, 1. The area of each peak represents the relative content of the corresponding substance.

### 2.6. Treatment of Data

Principal components analysis (PCA) and partial least squares discriminant analysis (PLS-DA) were conducted using metaX 1.4.16, a comprehensive software tool for metabolomics data processing. Statistical significance (*p*-value) was determined through univariate analysis (*t*-test). Metabolites that met the criteria of Variable Importance in Projection (VIP) > 1, *p*-value < 0.05, and fold change ≥ 2 or ≤0.5 were identified as differential metabolites. For clustering heat maps, the data were normalized using z-scores derived from the intensity areas of differential metabolites and visualized using the Pheatmap package in the R programming language. The correlation among differential metabolites was assessed using the cor() function in R, employing the Pearson method. Significance testing for correlations was conducted using the cor.mtest() function in R, with a significance threshold set at *p* < 0.05. Visualization of correlation plots was achieved through the corrplot package in R. The functions and metabolic pathways of these metabolites were analyzed using the KEGG database. Enrichment analysis of differential metabolites was based on a ratio threshold of x/n > y/N, with metabolic pathways deemed enriched when meeting this criterion. Additionally, metabolic pathways were considered statistically significantly enriched when the *p*-value was less than 0.05.

### 2.7. Antioxidant and Metabolite Correlation Network Diagram

Principal components analysis (PCA) and partial least squares discriminant analysis (PLS-DA) were conducted using metaX, a comprehensive software tool for metabolomics data processing.

## 3. Results

### 3.1. Antioxidant Activity Analysis of Different Varieties of M. chinensis Benth

**Figure 2:**
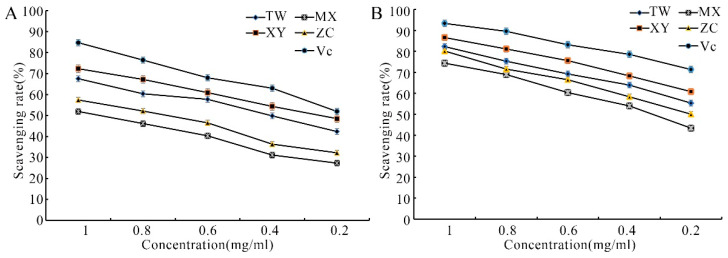
The scavenging rate of DPPH radicals and ABTS radicals of different *M. chinensis* Benth varieties. (**A**) The scavenging rate of DPPH radicals of different *M. chinensis* Benth varieties. (**B**) The scavenging rate of ABTS radicals of different *M. chinensis* Benth varieties.

With the decrease in extract concentration, the scavenging ability of different varieties of DPPH free radicals also decreased. At 1.0 mg/mL, the scavenging rates of TW, XY, MX, and ZC for DPPH free radicals were 67.6%, 72.3%, 51.9%, and 57.4%, respectively, and were all lower than that of Vitamin C (Vc) for DPPH free radicals at 84.7% (Figure 2A). Similarly, with the decrease in extract concentration, the scavenging ability of different varieties of ABTS free radicals also decreased. At 1.0 mg/mL, the scavenging rates of TW, XY, MX, and ZC for ABTS free radicals were 82.3%, 86.6%, 74.4%, and 80.1%, respectively, which were all lower than that of Vc for ABTS free radicals at 93.3% (Figure 2B). The values of half maximal inhibitory concentration (IC_50_) of half scavenging concentration of DPPH free radicals and ABTS free radicals for the four varieties and Vc are shown in Table 1. As can be seen, XY demonstrated the highest antioxidant capacity among the four varieties; however, it failed to surpass the activity level of the Vc positive control.

### 3.2. Determination of TPC, TFC, and MPC

The active components in *M. chinensis* Benth include antioxidants, total flavonoids, total phenolic acids, and polysaccharides [12,15]. The Total Phenolic Content (TPC), Total Flavonoid Content (TFC), and *M. chinensis* Benth Polysaccharides Content (MPC) for the four varieties are detailed in Table 2. TPC values varied between 52.40 ± 0.15 mg/g and 55.81 ± 0.84 mg/g. Variety XY displayed the highest TPC value at 55.81 ± 0.84 mg/g. TFC values ranged from 95.58 ± 4.20 mg/g to 126.18 ± 13.91 mg/g, with TW recording the highest TFC at 126.18 ± 13.91 mg/g. The MPC values varied significantly, ranging from 35.04 ± 2.68 mg/g to 114.19 ± 2.41 mg/g, with XY recording the highest MPC value at 114.19 ± 2.41 mg/g.

### 3.3. Detection Metabolites of M. chinensis Benth by Quasi-Targeted Metabonomics

Details of all identified metabolites are shown in Table S1. In total, 1287 metabolites were identified, including 193 amino acids and their derivatives, 182 flavonoids, 125 lipids, 116 organic acids and their derivatives, 108 carbohydrates and their derivatives, 93 organoheterocyclic compounds, 81 terpenoids, 70 nucleotides and their derivates, 70 phenolic acids, 59 phenylpropanoids and polyketides, 48 phenols and their derivatives, 43 alkaloids and derivatives, 32 amines, 21 phytohormones, 20 vitamins, 12 alcohols and polyols, 6 benzenes and substituted derivatives, 4 polyamines, 2 organosulfur compounds, and 2 organooxygen compounds.

### 3.4. Principal Component Analysis and Orthogonal Projections to Latent Structures Discriminant Analysis of Four Varieties M. chinensis Benth

**Figure 3:**
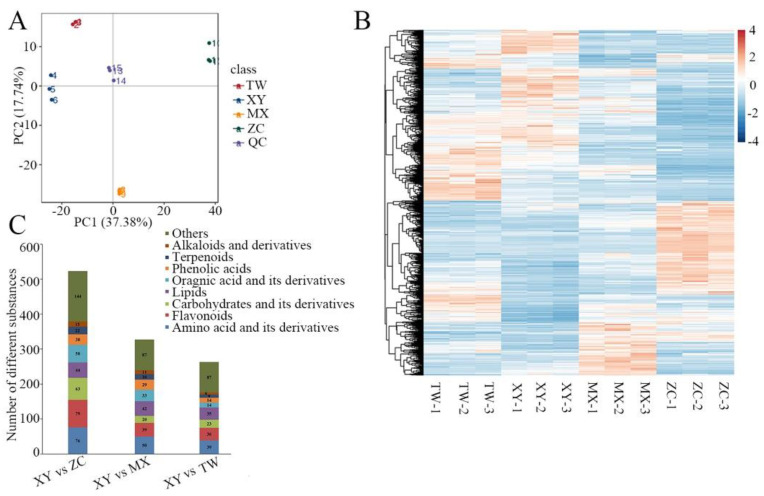
Overview of metabolites analysis in *M. chinensis* Benth. (**A**) Principal component analysis of metabolites levels in TW, XY, MX, and ZC. QC are samples used to test the accuracy, repeatability, and reliability of the analysis process. (**B**) Cluster heatmap depicting variations in metabolite content among samples. (**C**) Quantification of differentially abundant metabolites across various comparison groups.

Distinctions among the four varieties of *M. chinensis* Benth are discernible and confirmed through 2D PCA analysis (Figure 3A). The PCA score plot reveals that principal components PC1 and PC2 account for 37.38% and 17.74% of the total variance, respectively. The findings demonstrate a clear separation among the four varieties, with the three biological replicates of each variety forming closely clustered groups, thus indicating the reproducibility and validity of the study. This analysis revealed notable distinctions among the four varieties, with all samples falling within a 95% confidence interval. Furthermore, the cluster heat maps and corresponding metabolite heat maps demonstrate the consistency of components within biological replicates and the variability of components across different varieties (Figure 3B). Since XY is the most antioxidant variety, the differential metabolites of XY compared with the other three varieties were analyzed. In comparisons of XY vs. ZC, XY vs. MX and XY vs. TW, a total of 523, 327 and 263 secondary metabolites were identified, respectively. These metabolites were categorized into nine major types, with flavonoids, amino acids, and their derivatives being the predominant differentiating metabolites across all comparison groups (Figure 3C).

### 3.5. Sample Quality Control and PLS-DA Results

**Figure 4:**
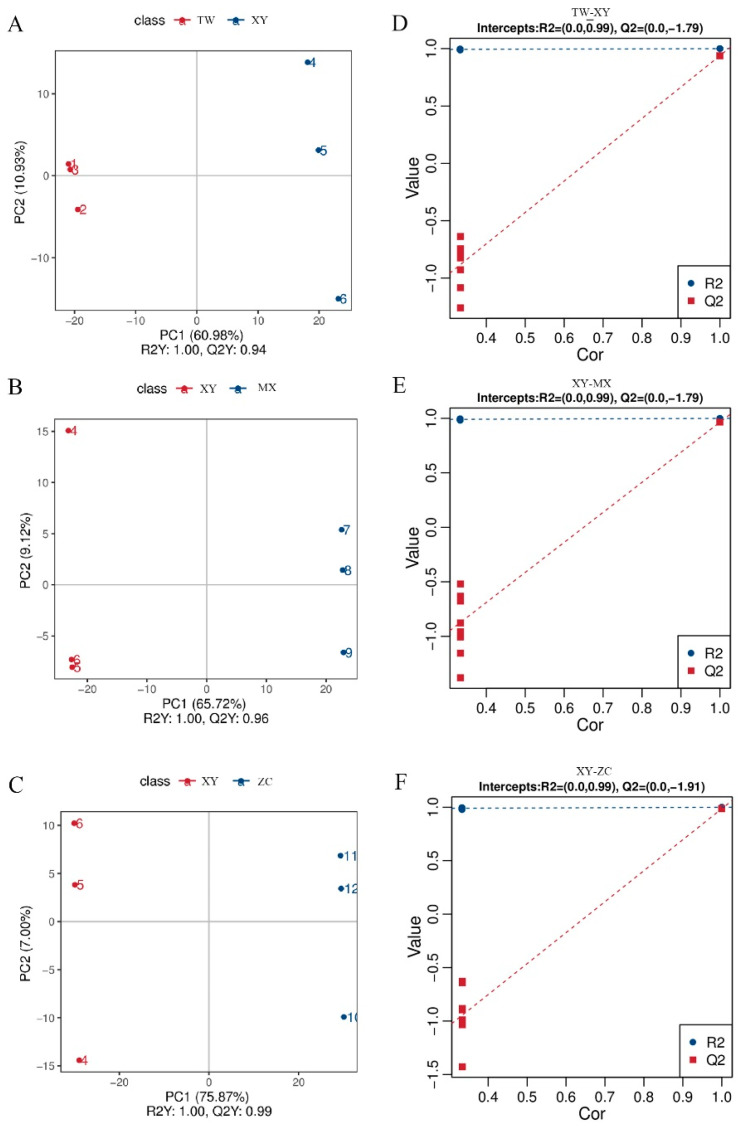
Partial least squares-discriminant analysis (PLS–DA) scores. Scores from the PLS–DA model comparing (**A**) XY vs. TW, (**B**) XY vs. MX, and (**C**) XY vs. ZC. PLS-DA S-plot model comparing (**D**) XY vs. TW, (**E**) XY vs. MX, and (**F**) XY vs. ZC. R2Y scores, and Q2 values assess the interpretative and predictive capabilities of the model, with Q2 > 0.5 indicating an effective model and Q2 > 0.9 indicating an excellent model.

Metabolomics data in both positive and negative modes were integrated for analysis. The use of partial least squares discriminant analysis (PLS-DA) facilitated a supervised multivariate statistical approach, enabling clear differentiation between age groups (Figure 4A–C). In cross-validation, R2Y and Q2Y values, which denote the model’s explanatory and predictive performance, respectively, exceeded 0.5, underscoring the robust interpretative and predictive capabilities of the models [16]. Permutation tests were subsequently conducted to assess and minimize the risk of overfitting (Figure 4D–F). The data indicated that the blue line (R2) consistently exhibited higher values than the red line (Q2), suggesting a reduced likelihood of overfitting [17].

### 3.6. Differential Metabolite Screening, Functional Annotation, and Enrichment Analysis of XY Compared to the Other Three Varieties

**Figure 5:**
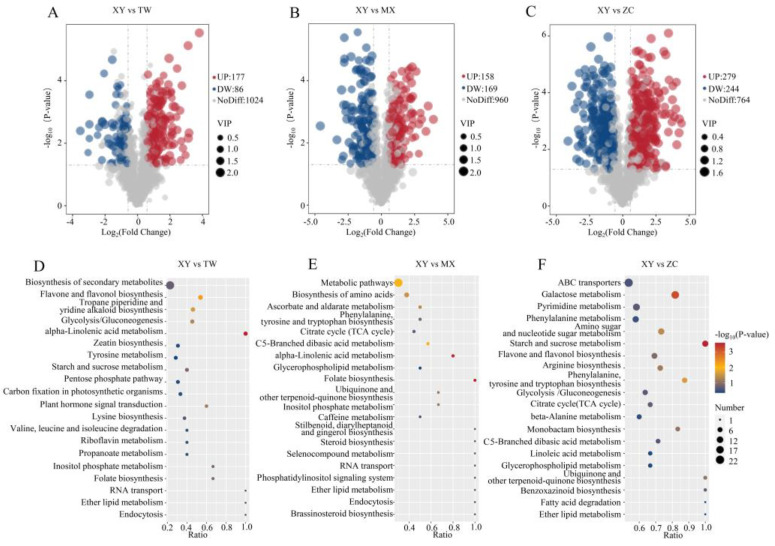
Volcano maps and KEGG enrichment maps of differential metabolites among three comparison groups. (**A**–**C**) Volcano maps of differential metabolites in TW vs. XY, XY vs. MX, and XY vs. ZC. (**D**–**F**) KEGG enrichment maps of differential metabolites in TW vs. XY, XY vs. MX, and XY vs. ZC.

Based on a fold change of ≥2 or ≤0.5 and a VIP of ≥1, differential metabolites were identified in pairwise comparisons, as shown in Figure 5A–C. Among these, the XY vs. TW comparison exhibited the fewest differential metabolites with 263 identified (177 upregulated and 86 downregulated). Furthermore, 327 significant differences were observed between XY and MX (158 upregulated, 169 downregulated) and 523 between XY and ZC (279 upregulated, 244 downregulated). An intersection analysis using a Venn diagram revealed that only 56 metabolites were common across the comparison groups (XY vs. TW, XY vs. MX, and XY vs. ZC) (Figure S1). In this study, KEGG analysis was also performed on the differential metabolites of XY vs. TW, XY vs. MX, and XY vs. ZC, and the top 20 pathways with the highest enrichment were shown in Figure 5D–F. These include a variety of amino acid metabolic pathways and secondary metabolic pathways (flavone and flavonol biosynthesis, Stilbenoid, diarylheptanoidand gingerol biosynthesis, Phenylalanine metabolism). These results may reveal the reason why XY varieties have higher TPC and TFC content and stronger antioxidant activity.

Using the expression level of significantly different metabolites, the samples of different groups were stratified and clustered. The results of hierarchical cluster analysis (HCA) showed that the metabolites of markers between XY and the other three varieties were mainly composed of amino acids and their derivatives, flavonoids, and phenolic acids (Figures S2–S4).

### 3.7. Correlation Analysis of Secondary Metabolites in M. chinensis Benth and Their Antioxidant Activity

**Figure 6:**
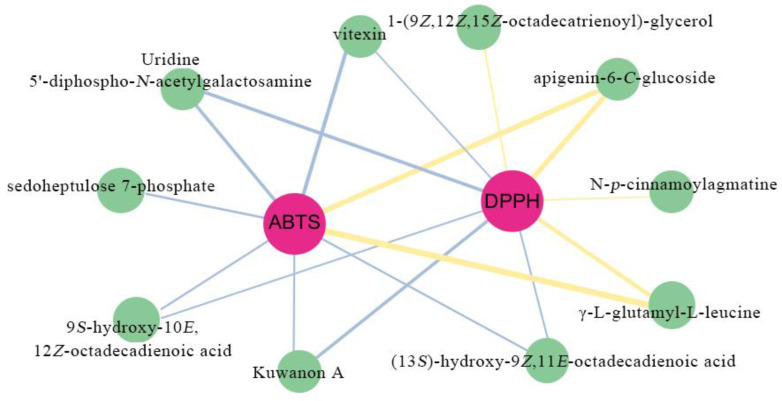
Network diagram between antioxidant capacity and metabolites. Pink circles represent antioxidant capacity, specifically the ability to scavenge the DPPH and ABTS radical. Green circles represent various metabolites. The blue line signifies a negative correlation and the yellow line signifies a positive correlation, with a correlation coefficient of r ≥ 0.8 and a significance level of *p* < 0.05. The thickness of the lines corresponds to the magnitude of the correlation coefficient |rho|, with thicker lines indicating stronger correlations.

To further investigate the principal compounds influencing the elevated antioxidant activity of XY, we conducted a screening of the common differential metabolites across three comparative groups: XY vs. MX, XY vs. ZC, and XY vs. TW. The screening criteria were set at |rho| > 0.8 and *p* < 0.05, resulting in the identification of 10 substances. Subsequently, network diagrams illustrating the correlations between these 10 metabolites and the DPPH and ABTS assays were constructed utilizing Spearman correlation analysis. In these, three flavonoids (apigenin-6-*C*-glucoside, Kuwanon A, vitexin), three lipids (9*S*-hydroxy-10*E*,12*Z*-octadecadienoic acid, (13*S*)-hydroxy-9*Z*,11*E*-octadecadienoic acid,1-(9*Z*,12*Z*,15*Z*-octadecatrienoyl)-glycerol), one nucleotide and its derivates (Uridine 5′-diphospho-*N*-acetylgalactosamine), one phenolic acid (*N*-*p*-cinnamoylagmatine), one carbohydrate and its derivatives (sedoheptulose 7-phosphate), and one amino acid and its derivatives (γ-L-glutamyl-L-leucine) were significantly correlated with antioxidant capacity, indicating that in addition to flavonoids, lipids, nucleotides and their derivates, phenolic acids, carbohydrates and their derivatives, and amino acids and their derivatives may also be important antioxidants (Figure 6).

## 4. Discussion

### 4.1. Variability in Secondary Metabolites Among M. chinensis Benth Varieties

Varieties refer to regional adaptive varieties originating from distinct natural distribution areas or geographically isolated varieties resulting from artificial cultivation and domestication. Variations in growth, development, and quality among different varieties form the basis for the selection and breeding of superior varieties [13]. Previous research on the metabolites of *M. chinensis* Benth is limited. In this study, we conducted a comprehensive metabolome analysis of four *M. chinensis* Benth varieties, providing a detailed metabolic profile of the plant. This study demonstrated that XY varieties exhibited the highest total antioxidant activity, with flavonoids and polyphenols serving as significant contributors to their antioxidant efficacy. Past studies have revealed the antioxidant capacity of *M. chinensis* Benth [12,15]. By using UPLC-Q-TOF-MS/MS technology, concentrating on polyphenols and terpenoids, and successfully identified 57 compounds [12]. Eight major compounds were identified through routine analysis using HPLC-MS technology [16]. In contrast, the present study utilized UHPLC-MS/MS techniques to identify a more extensive array of metabolites, detecting a total of 1287, which included various amino acids, lipids, and organic acids. This paper represents the first systematic and comprehensive analysis of the metabolite composition of *M. chinensis* Benth and its relationship with antioxidant activity.

Significant differences were observed in the content of kaempferol 3-O-robinbioside among the native varieties TW, XY, MX, and ZC when comparing the types and concentrations of various metabolites. The highest content of kaempferol 3-O-robinbioside was found in variety XY, a flavonoid compound known to significantly inhibit human lymphocyte proliferation in vitro [16]. Similarly, the composition of vitexin varies significantly, with vitexin demonstrating antioxidant properties against reactive oxygen species, lipid peroxidation, and other forms of oxidative damage in various oxidative stress-related diseases [17]. Additionally, the composition of nodakenin also shows significant variations, with nodakenin exhibiting anti-inflammatory, antibacterial, antioxidant, and anti-platelet aggregation effects [18]. Therefore, these metabolites serve as indicators for assessing the quality of *M. chinensis* Benth. Notably, caffeic acid exhibits antiviral properties in vitro, with MX showing the highest caffeic acid content among the four varieties [19]. Furthermore, triterpene acids in *M. chinensis* Benth display anti-inflammatory effects [20], with consistent detection of triterpene acids across all four varieties, albeit at similar content levels.

### 4.2. Relationship Between Composition and Antioxidant Capacity of Secondary Metabolites in M. chinensis Benth

This study has identified 10 distinct metabolites in *M. chinensis* Benth that exhibit a significant correlation with antioxidant activity. These include flavonoids, lipids, nucleotides and their derivates, phenolic acids, and carbohydrates and their derivatives. The findings underscore that flavonoids, in particular, possess potent antioxidant properties due to their rich hydroxyl group content. By scavenging free radicals, flavonoids can effectively mitigate oxidative damage and potentially reduce the risk of chronic diseases, such as cardiovascular diseases [21]. Many phenolic acids also exhibit antioxidant properties; for example, gallic acid is a potent constituent of *Paeonia rubra* radix Hort ex Steud in traditional Chinese medicine, known for its robust antioxidant activity [22]. Phenolic compounds, acting as effective electron donors, can serve as antioxidants, exemplified by salvianolic acid B, a highly potent natural product renowned for its antioxidant properties [23]. Previous research suggests that polyphenols, including caffeic acid and kaempferol, may play a significant role in the antioxidant properties of *M. chinensis* Benth [24,25]. However, our study found that while flavonoid compounds constituted the largest proportion of total metabolites, the total phenolic acid content did not demonstrate a significant positive correlation with antioxidant activity, contrary to some prior findings. Additionally, polysaccharides from *M. chinensis* Benth exhibited notable antioxidant properties [26,27], with the highest levels of XY polysaccharide content and DPPH free radical clearance rate observed in this study. Our research also revealed significant variations in the levels of ten metabolites such as vitexin among four distinct varieties, compounds not previously reported in *M. chinensis* Benth. Further investigation is warranted to explore the separation, purification, and biological functions of these noteworthy compounds.

## 5. Conclusions

This study represents the first systematic investigation into the secondary metabolic composition and antioxidant capacity of *M. chinensis* Benth across four distinct varieties (TW, XY, MX, and ZC). A total of 1287 metabolites, including flavonoids and phenolic acids, were identified. Differentially accumulated metabolites were found to be enriched in secondary metabolic pathways such as “Tropane, piperidine, and pyridine alkaloid biosynthesis” and “Flavone and flavonol biosynthesis”. Significant variations in metabolite quantities were observed among the four *M. chinensis* Benth varieties, with XY exhibiting the highest TFC and antioxidant activity. Correlation analysis revealed that 10 distinct metabolites showed a significant correlation with antioxidant capacity, with flavonoids and lipids emerging as the primary contributors to variations in antioxidant activities. This research provides valuable insights for enhancing breeding programs, quality assurance measures, and the development of products derived from *M. chinensis* Benth.

## Figures and Tables

**Table 1:** Antioxidant activity analysis of *M. chinensis* Benth.

Materials	IC_50_ (mg/mL)	
	DPPH Radical Scavenging Effects	ABTS Radical Scavenging Effects
TW	0.36 ± 0.01	0.17 ± 0.02
XY	0.25 ± 0.01	0.13 ± 0.01
MX	1.02 ± 0.02	0.31 ± 0.03
ZC	0.72 ± 0.02	0.23 ± 0.01
Vc	0.20 ± 0.01	0.08 ± 0.00

Note: Values are presented as mean and pooled standard deviation (n = 3); values are presented as the mean ± standard deviation (SD), calculated on a dry basis. The data were retained to two decimal places and subsequently rounded.

**Table 2:** Active ingredient content of *M. chinensis* Benth.

Materials	TPC (mg/g)	TFC (mg/g)	MPC (mg/g)
TW	54.56 ± 0.61 a	126.18 ± 13.91 a	44.78 ± 1.20 b
XY	55.81 ± 0.84 a	125.88 ± 7.66 a	114.19 ± 2.41 a
MX	52.50 ± 0.39 b	95.58 ± 4.20 b	46.49 ± 0.81 b
ZC	52.40 ± 0.15 b	100.81 ± 3.06 b	35.04 ± 2.68 c

Note: TPC, total phenolic acid content. TFC, total flavonoid content. MPC, *M. chinensis* Benth polysaccharides. Values are presented as mean and pooled standard deviation (n = 3); different lowercase letters indicate significant differences (*p* < 0.05) according to Duncan’s test. For example, the TPC value is not significantly different between TW (a) and XY (a), but MX (b) is significantly different from TW (a) and XY (a). Values are presented as the mean ± standard deviation (SD), calculated on a dry basis.

## Data Availability

Data are contained within the article or Supplementary Materials.

## Supplementary Materials

The following supporting information can be downloaded at: https://www.mdpi.com/article/10.3390/plants14111585/s1. Figure S1: Venn diagram analysis of differential metabolites in three control groups (XY vs. TW, XY vs. MX, and XY vs. ZC. Figure S2: Heatmaps analysis of differential metabolites in XY vs. TW. Figure S3: Heatmaps analysis of differential metabolites in XY vs. MX. Figure S4: Heatmaps analysis of differential metabolites in XY vs. ZC. Table S1. Metabolites in all samples. Table S2. The multiple of difference in the content of 24 substances in the three comparison groups. Table S3. Correlation calculation result.
