# Supplementary figures and images for: Quasi-Targeted Metabonomics Reveals Metabolites Associated with Antioxidant Activity of Mesona chinensis Benth Cultivar Xiaoye
Source: Plants (Basel). 2025 May 23;14(11):1585. doi: 10.3390/plants14111585 (PMC12158099; doi:10.3390/plants14111585)

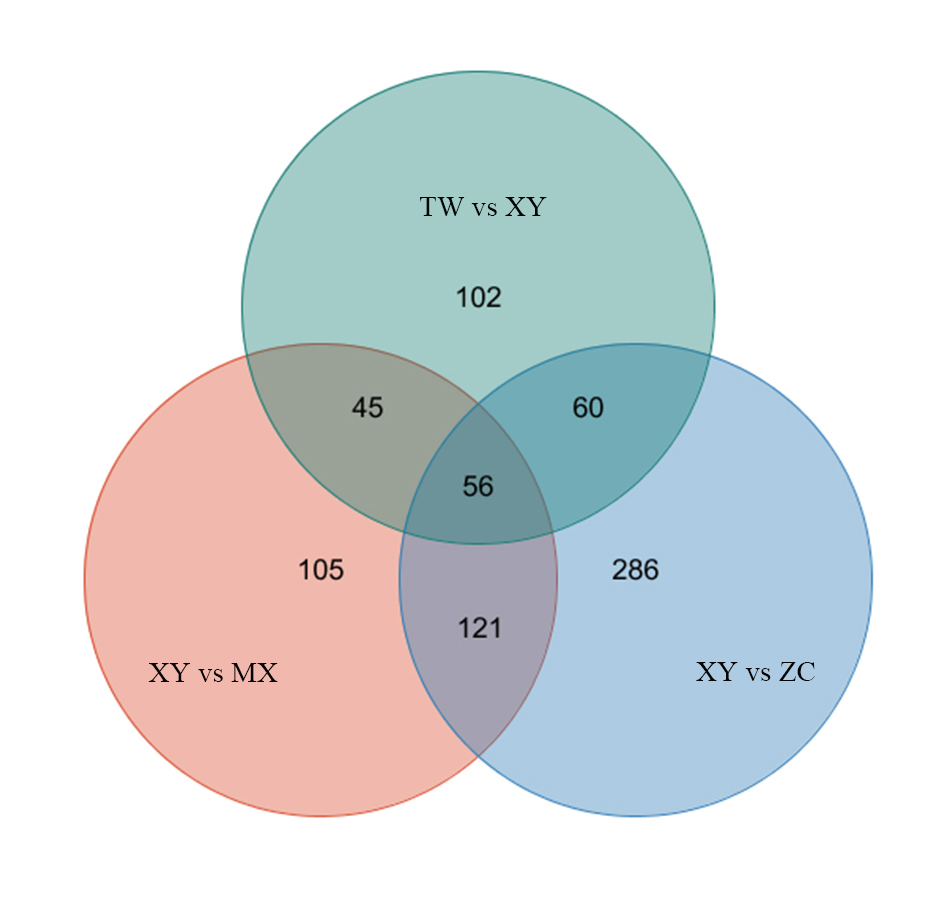

Supplement: Supplementary file 1 [file plants-14-01585-s001.zip › Figure S1.jpg]

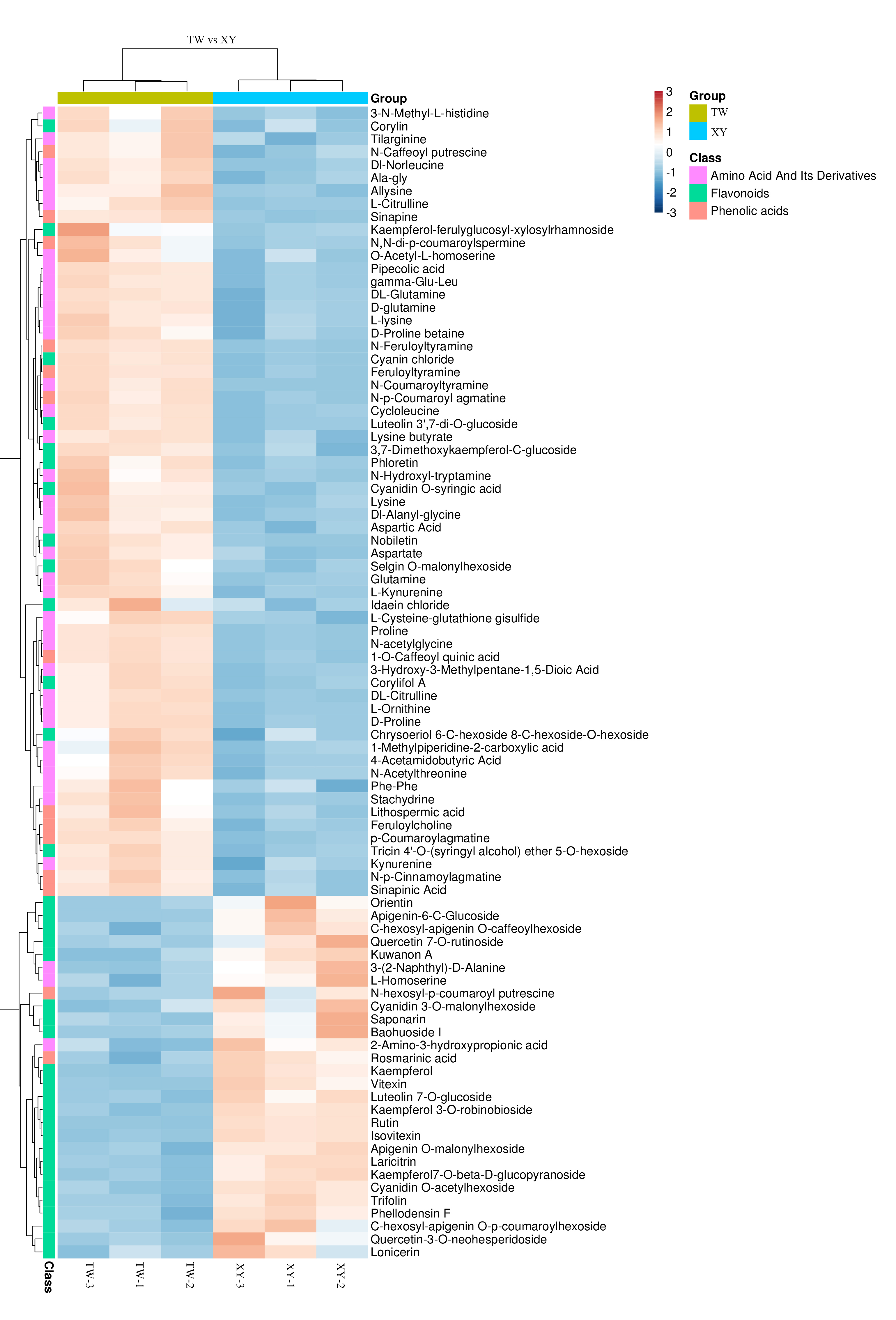

Supplement: Supplementary file 1 [file plants-14-01585-s001.zip › Figure S2.jpg]

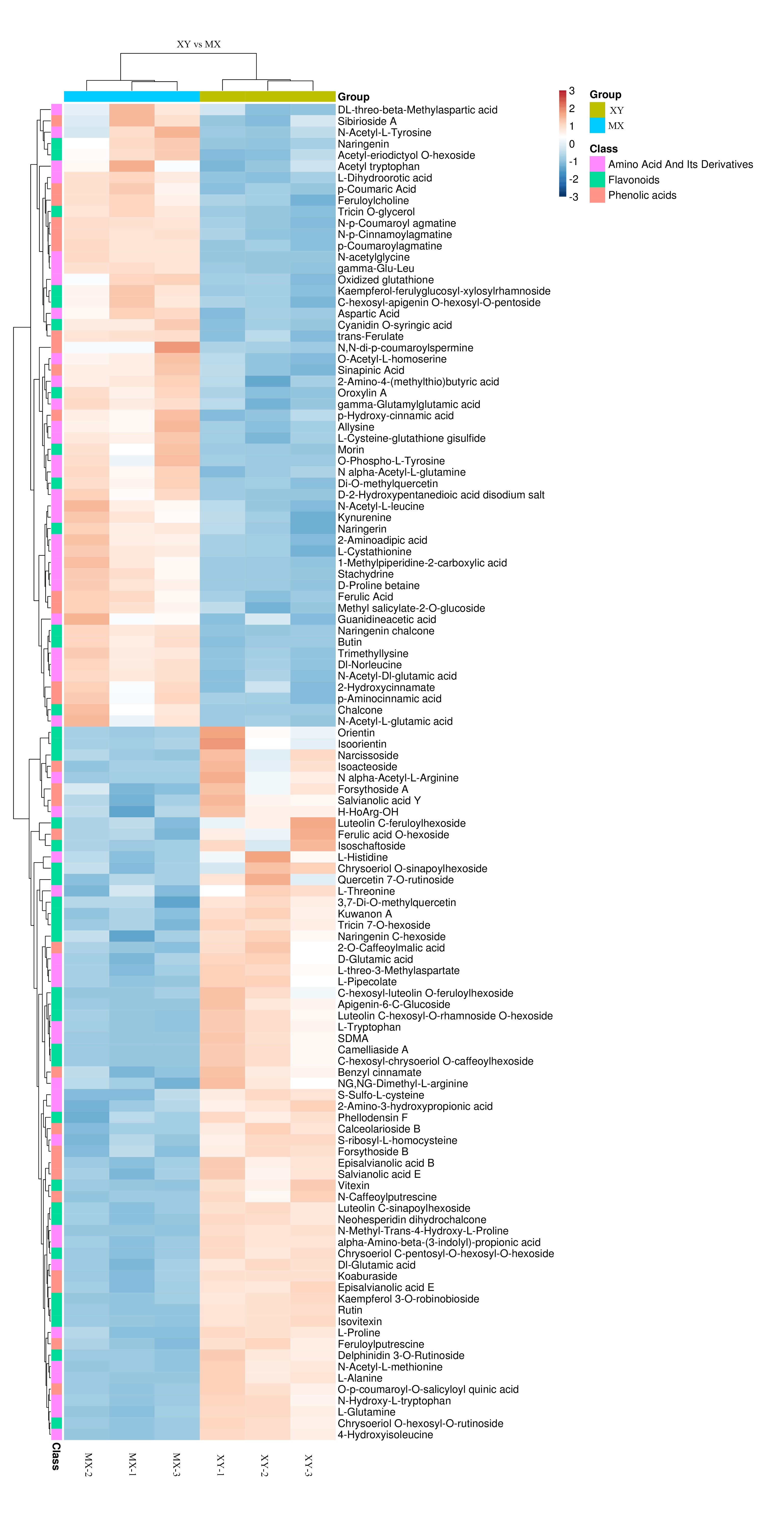

Supplement: Supplementary file 1 [file plants-14-01585-s001.zip › Figure S3.jpg]

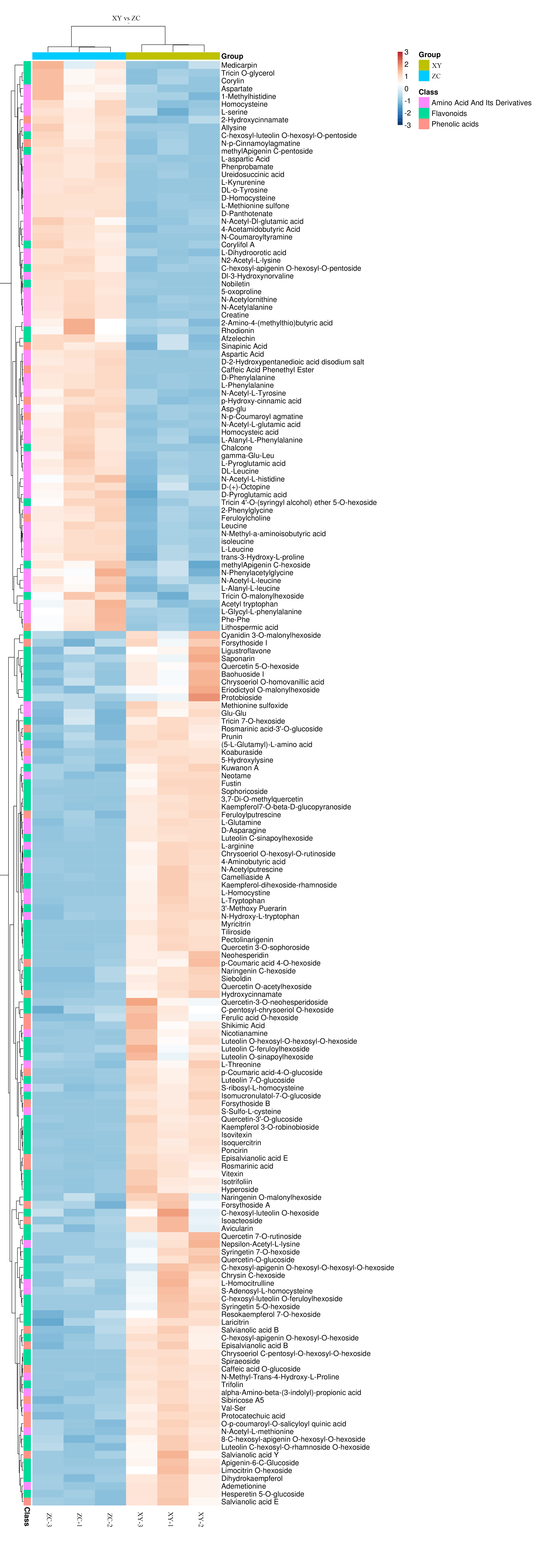

Supplement: Supplementary file 1 [file plants-14-01585-s001.zip › Figure S4.jpg]
